# The role of artificial intelligence in pandemic responses: from epidemiological modeling to vaccine development

**DOI:** 10.1186/s43556-024-00238-3

**Published:** 2025-01-03

**Authors:** Mayur Suresh Gawande, Nikita Zade, Praveen Kumar, Swapnil Gundewar, Induni Nayodhara Weerarathna, Prateek Verma

**Affiliations:** 1 Department of Artificial Intelligence and Data Science, Faculty of Engineering and Technology, Datta Meghe Institute of Higher Education and Research (Deemed to Be University), Sawangi (Meghe), Wardha, Maharashtra 442001 India; 2 Department of Computer Science and Medical Engineering, Faculty of Engineering and Technology, Datta Meghe Institute of Higher Education and Research (Deemed to Be University), Sawangi (Meghe), Wardha, Maharashtra 442001 India; 3 Department of Artificial Intelligence and Machine Learning, Faculty of Engineering and Technology, Datta Meghe Institute of Higher Education and Research (Deemed to Be University), Wardha, Maharashtra 442001 India; 4 Department of Biomedical Sciences, School of Allied Health Sciences, Datta Meghe Institute of Higher Education and Research (Deemed to Be University), Wardha, Maharashtra 442001 India

**Keywords:** Artificial intelligence, COVID-19, Global health, Epidemiological modelling, Machine learning algorithms, Vaccine development, SARS-CoV-2

## Abstract

Integrating Artificial Intelligence (AI) across numerous disciplines has transformed the worldwide landscape of pandemic response. This review investigates the multidimensional role of AI in the pandemic, which arises as a global health crisis, and its role in preparedness and responses, ranging from enhanced epidemiological modelling to the acceleration of vaccine development. The confluence of AI technologies has guided us in a new era of data-driven decision-making, revolutionizing our ability to anticipate, mitigate, and treat infectious illnesses. The review begins by discussing the impact of a pandemic on emerging countries worldwide, elaborating on the critical significance of AI in epidemiological modelling, bringing data-driven decision-making, and enabling forecasting, mitigation and response to the pandemic. In epidemiology, AI-driven epidemiological models like SIR (Susceptible-Infectious-Recovered) and SIS (Susceptible-Infectious-Susceptible) are applied to predict the spread of disease, preventing outbreaks and optimising vaccine distribution. The review also demonstrates how Machine Learning (ML) algorithms and predictive analytics improve our knowledge of disease propagation patterns. The collaborative aspect of AI in vaccine discovery and clinical trials of various vaccines is emphasised, focusing on constructing AI-powered surveillance networks. Conclusively, the review presents a comprehensive assessment of how AI impacts epidemiological modelling, builds AI-enabled dynamic models by collaborating ML and Deep Learning (DL) techniques, and develops and implements vaccines and clinical trials. The review also focuses on screening, forecasting, contact tracing and monitoring the virus-causing pandemic. It advocates for sustained research, real-world implications, ethical application and strategic integration of AI technologies to strengthen our collective ability to face and alleviate the effects of global health issues.

## Introduction

The sudden surge in widespread fatalities jolted the world out of its complacency. Initial reports emerging from Wuhan, located in China’s Hubei Province, back in December 2019, hinted at symptoms akin to pneumonia, signaling the presence of a virus [1–3]. Early investigations pointed toward individuals who frequented the Huanan Seafood Wholesale Market. However, cases where patients denied contact with the market underscored the virus’s alarming ability for human-to-human transmission. Subsequent efforts by the Chinese Center for Disease Control and Prevention (CDC) and health authorities pinpointed a novel coronavirus as the culprit behind the pneumonia-like outbreak in Wuhan, subsequently termed COVID-19, or coronavirus disease 2019. This virus was identified as the severe acute respiratory syndrome coronavirus. The World Health Organization (WHO) swiftly declared a Public Health Emergency of International Concern in response to COVID-19, as per data obtained from the “Coronavirus COVID-19 Global Cases” dashboard by the Center for Systems Science and Engineering (CSSE) at Johns Hopkins University (JHU) [4, 5]. For diagnosis, the WHO recommended using RT-PCR tests for suspected COVID-19 cases. Currently, until August 2024, over 775.45 million confirmed cases have been documented across 189 countries, with a staggering death toll surpassing 7.1 million worldwide. Despite ongoing clinical trials, no definitive medication or vaccine for COVID-19 remains. The WHO officially declared the outbreak a Public Health Emergency of International Concern on January 30, 2020, and later christened the disease COVID-19 in February of the same year, with “COVID” standing for “CO” (corona), "VI" (virus), “D” (disease), and “2019” denoting the year of its emergence [4, 6, 7].

The primary causative agent of COVID-19 is SARS-CoV-2, which shares 79.6% genomic similarity with SARS-CoV and exhibits a 96% genetic similarity with SAR virus identified in bats [8, 9]. Studies show that the median incubation period for COVID-19 is approximately three days, from 0 to 24 days, and transmission may occur in asymptomatic individuals. This outbreak represents the sixth declaration of a Public Health Emergency of International Concern (PHEIC), following previous global health events such as the H1N1 influenza pandemic in 2009, the polio resurgence in 2014, the Ebola virus outbreaks in West Africa in 2014 and the Democratic Republic of the Congo in 2019, and the Zika virus epidemic in 2016 [10, 11].

Many countries enacted stringent lockdowns and rigorous screening measures at international borders in response to the pandemic. The global economic impact of these measures, often called “The Great Lockdown,” is projected to result in the most severe financial crisis since the Great Depression. In the United States, unemployment rates were forecasted to rise sharply, potentially reaching 14%, with some estimates predicting levels as high as 20% in the aftermath of the pandemic. Similar economic disruptions have been observed across Europe and the Americas, with multiple sectors facing layoffs and financial losses.

In biotechnology, Artificial Intelligence (AI) is recognised as playing a crucial role in combating the virus. AI could expedite the development of treatments and solutions. As observed in recent studies, the pandemic has spurred increased interest and utilisation of AI and data analytics across various fields [12]. AI has become a broad field encompassing multiple domains, such as machine learning, deep learning, robotics, natural language processing, vision and sensory systems, expert systems, and decision aids. Generally, AI refers to a machine’s ability to learn from experience, adapt to new inputs, and perform tasks akin to human capabilities. Due to the evolving nature of this technology, there is yet to be a universally accepted definition of AI. AI relies heavily on vast datasets to train pattern recognition, which may not make it the most suitable tool for detecting novel pandemics. However, AI can be pre-trained on data related to similar diseases, such as MERS or SARS, to aid in identifying emerging threats like COVID-19. Early detection and forecasting of pandemics offer crucial time to bolster supplies, preparedness, and research efforts [10, 13–19]. Regrettably, there have been instances where human decision-makers overlooked AI alerts and warnings from virologists, highlighting that AI is merely a tool at the disposal of humans. Disregarding warnings from such advanced tools can lead to detrimental consequences [20–22].

The data presented shows that the integration of AI in pandemic management has significantly influenced the number of survivors over time. In this context, epidemiological modelling emerges as a crucial component, leveraging machine learning methodologies to analyze extensive datasets comprising demographic details, travel behaviours, healthcare records, and environmental variables. These models predict disease transmission patterns, pinpoint vulnerable populations, and assess the effectiveness of various intervention strategies. Epidemiologists frequently employ sophisticated computational tools to simulate hypothetical scenarios, evaluate the impact of public health policies, and provide informed recommendations to policymakers in curtailing the spread of infectious diseases, including pandemics. AI has played a pivotal role throughout the pandemic response, contributing to early warnings, epidemiological modelling, and the expedited development of life-saving vaccines [23–29].

Reflecting on the insights gained during this unprecedented period, it’s evident that AI has emerged as a crucial ally in the global battle against infectious diseases, owing to its rapid data processing capabilities and ability to extract invaluable insights from vast datasets. This review delves into how the emergence of AI and epidemiological modelling, coupled with advanced AI tools, machine learning, and deep learning techniques, have bolstered survival rates during severe pandemics like COVID-19. Additionally, our study explores the intricate processes involved in vaccine development, clinical trials, vaccine distribution, and the ethical considerations imperative during a pandemic. However, it’s essential to acknowledge that ethical considerations must be noticed while AI is effective. This paper also delves into the moral frameworks and strategies guiding AI development amid pandemics, ensuring responsible and equitable utilisation of AI technologies in public health crises along with the challenges in collaborating AI and the prospects related to AI in pandemics like COVID-19 [30, 31].

## Epidemiological modeling

Throughout the pandemic, epidemic modelling has been extensively employed to assess the hazards posed by COVID-19, predict its impact on healthcare systems, elucidate the necessity for and advocate the adoption of preventive measures, optimise the distribution of vaccinations, and influence various aspects of daily life. "Epidemic modelling" encompasses a range of methodologies for analysing the transmission of infectious diseases within populations, employing computer simulations, statistical analyses, and mathematical techniques. It draws upon information and theories to elucidate disease transmission dynamics, population dynamics over time, and their effects on public health [32–35].

Epidemiology, a discipline that has its roots in the study of what happens to a population, has been defined by John Last in the Dictionary of Epidemiology as “the study of the distribution and determinants of health-related states or events in specified populations, and the application of this study to the control of health problems”. Epidemiologists are concerned with the disease and “health-related events”; ultimately, epidemiology is committed to disease control [36, 37]. As such, epidemiology constitutes an essential discipline of public health since it aims to quantify the health status of populations to identify its causes and propose interventions to improve the health of the population concerned and to evaluate them.

**Fig. 1:**
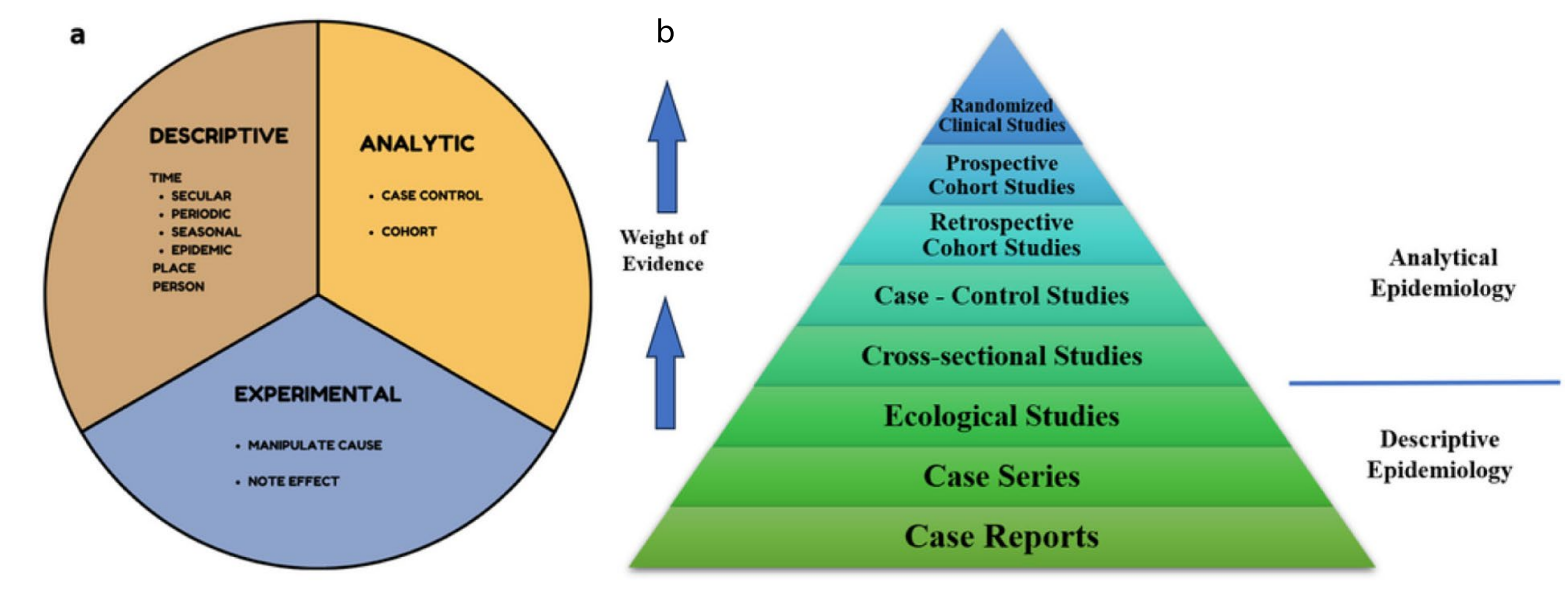
**a** Schematic representation of the types of Epidemiology. **b** Evidence Hierarchy of Epidemiological Study Designs how the flow of epidemiological studies tends from initial to final stage

The bare brick of epidemiology remains the data, which must be as valid and precise as possible to ensure they measure what they are supposed to measure (validity) and limit measurement error (reliability). Evidence in epidemiology is based on the standardised production of data, specifying the volume and scope of the data to be collected [37–40]. The logic consists of working with a limited set of precisely characterized, traced, and even certified data. It is a question of specifying a set of criteria upstream of the investigation to represent the population studied and the values investigated and ensure that the measures will likely respect its characteristics. The nature of the questions to be answered and the methods used to answer them are the defining characteristics of the various fields of epidemiology. Traditionally, a distinction has been established between i) descriptive epidemiology, which aims to characterise the incidence and distribution of health factors or phenomena in populations based on human, geographical, and temporal features. Here, the goal is to estimate surveillance indicators like survival, incidence, and prevalence. ii) analytical epidemiology seeks to determine and quantify the relationship between exposures to particular variables and the eventual occurrence of illness or health events. Iii) evaluative epidemiology, which attempts to assess the efficacy of a health program or policy intervention on the illness or health condition under investigation, and the search for determinants to analyse the patterns and the causes [41–43]. Figure 1 shows the representation of types of Epidemiology and the hierarchy of Epidemiologic study designs.

Using mathematical methods to forecast results, analytical modelling offers theoretical insights and broad applicability but may need sophisticated mathematical knowledge and frequently assumes ideal circumstances. Though it has difficulty forecasting future occurrences and establishing casualty, descriptive modelling helps understand present behaviours using observational data. It is also easy to use and excellent for identifying patterns. Although experimental modelling provides empirical data, it is frequently labour and resource-intensive and susceptible to ethical and practical limitations. It uses controlled simulations to test hypotheses and determine causation [44–46].

In population epidemiology, data can come from trial registries or cohorts and from data routinely collected in different systems, such as medico-administrative data, administrative data, and environmental data [47–49]. However, these data have limitations, such as needing to be constructed to study the health of populations, raising questions about their validity & reliability. Access to these databases, particularly at the regulatory level, is also an issue. Epidemiology has always used numerical methods to produce results, whether describing, understanding, or evaluating parameters hidden from observation. These include three basic epidemiological models, namely, SIS endemic, SIR epidemic and SIR endemic, which helps in experimentation. The definition, collection, and analysis of data are at the heart of epidemiology, making it a discipline particularly concerned with the development of what is known as massive data and AI. Big data is the rapid processing of massive heterogeneous data that calls upon approaches, some of which are part of what is known as AI [50, 51]. These massive data opportunities provide opportunities to describe better, understand, and intervene in the health of populations, making them a tool for epidemiology and its development. The Role of AI in Epidemiology is explained and elaborated below.

## Role of AI in epidemiology

AI is revolutionising the field of epidemiology by providing advanced methods for observational data analysis. These methods, which can be compared to more classical methods, aim to identify associations between exposure and health outcomes. The abundance of data, diverse collection formats, and real-time nature of data make AI methods a natural reference for analyses that differ significantly from traditional epidemiology. Additionally, AI methods are often used in the analysis stage, where the diversity of data sources, types, and distribution in systems requires methods upstream of the analyses. These AI methods enable data collection, extraction, and structuring according to different models, allowing epidemiologists to use data for their research. Overall, AI is revolutionizing the field of epidemiology by providing a more comprehensive and efficient approach to data analysis. The following subsections provide an overview of AI technologies created for forecasting, social control and epidemiological prediction in the fight against COVID-19 [52–55]. The illustration of the use of AI for the organisation of data, the surveillance of diseases and health outcomes and finally, the use of data and model-driven approaches in Epidemiology is carried out below.

### AI for collecting, classifying and structuring the data

The fundamental components of epidemiology, as well as any general model or AI learning, are data. To train AI algorithms that will be helpful for epidemiology, it is therefore essential to be able to both collect and annotate data and employ the AI algorithms to aid in data gathering. EHRs, medico-administrative records, credit card transactions, social media, wearable and ambient sensors, mobile phones, and internet searches are just a few of the data sources used for public health monitoring in pandemics like COVID-19. In particular, where anonymous and probabilistic matching is required, AI may match data from several databases [56–58]. Electronic health records (EHR), cohort databases, medico-administrative databases, and open data can be used. Robust algorithm learning and distributed data are two challenges addressed by a specific use of AI called federated learning. AI can harmonise disparate sources or formats into a standard, organised model like EHR [59, 60]. A joint research framework is made possible by adopting interoperability standards as a supplemental or intermediary layer over current data sources. As an example, consider the OMOP initiative’s shared data model. Federated learning disperses training data to several sources, like each participating hospital, making AI algorithms suitable for epidemiology training possible. Other applications of this method beyond EHR include medication side effect prediction. The majority of the challenges that arise while learning AI from dispersed sources are solved by federating learning, and the results show that the algorithm’s performances are nearly identical to those from a centralised database [61, 62].

### AI-powered virtualization or reconstruction of experiments

When multivariable models are used in epidemiology to analyse observational data, they often reduce the impact of confounding variables to the greatest extent feasible, approaching the controlled experimental design observed in clinical trials. Through the use of sophisticated analysis and models, we attempt to retrieve what we were unable to enforce throughout the data-collecting process [63, 64]. AI holds the potential to create databases that almost replicate the conditions of a controlled experimental design by leveraging vast volumes of data. Virtual randomised controlled trials are what is discussed. For the time being, these methods are theoretical [65, 66].

### Surveillance of disease and health outcomes

Public health surveillance involves systematically collecting, analysing, and interpreting data to prevent and control diseases and injuries. The rapid development of data science, including big data and AI, has enabled health authorities to respond quickly to health crises [67, 68]. The access and use of data generated from hospitals and medical facilities provides an opportunity to understand, predict, and combat diseases at unprecedented speed. The COVID-19 pandemic has shown the importance of data in controlling the epidemic. Public health surveillance will use this approach in addition to existing systems for infectious and chronic diseases [69, 70].

### Data-driven knowledge and decision-making in epidemiology: from data to decision

The emergence of AI in various fields raises the question of what tasks will be automated and to what extent. AI will act as a substitute for humans when solving pandemics like COVID-19. An optimistic view of AI’s use aligns with industrialisation, automation, and the robotisation of assembly lines, allowing humans to focus on tasks specific to their condition. In epidemiology, AI can intervene at all stages, from data collection to decision-making [71]. AI can intervene in data collection, reconciliation, and structuring, particularly under conditions that would make it difficult for humans to access data in a reasonable time and with the right resources. It can also intervene in data analysis as a complementary technique to traditional methods. AI can also be placed further in the value-added chain of data processing, such as the automated choice of “best” models and predictors, monitoring an entire part of the healthcare system, and helping in public health management decisions based on collected data [72, 73].

The first use case is similar to 4P medicine, where AI can predict the occurrence of diabetes and cardiovascular diseases. Some authors highlight the need for human operators to effectively mix and synthesize large datasets, such as the Global Burden of Disease study. AI then screens all data and draws attention to potentially interesting results. More ambitious projects and systems promise projections over several decades in terms of the needs and use of the health system. Authors have produced 40-year projection models for Singapore, considering factors such as ethnicity, social isolation, or disability [74, 75]. Others have developed and calibrated an AI called automated time series machine learning, applying it to Romanian data to predict the population’s health over the coming years about the ten most common diseases. Some authors have taken the reasoning to the extreme, sweeping aside causal and explanatory models of diseases and health events for statistical inference and controlled experiments. This vision would be the “end of the theory” in epidemiology, investigating the causes of population health states [4, 53, 76].

**Fig. 2:**
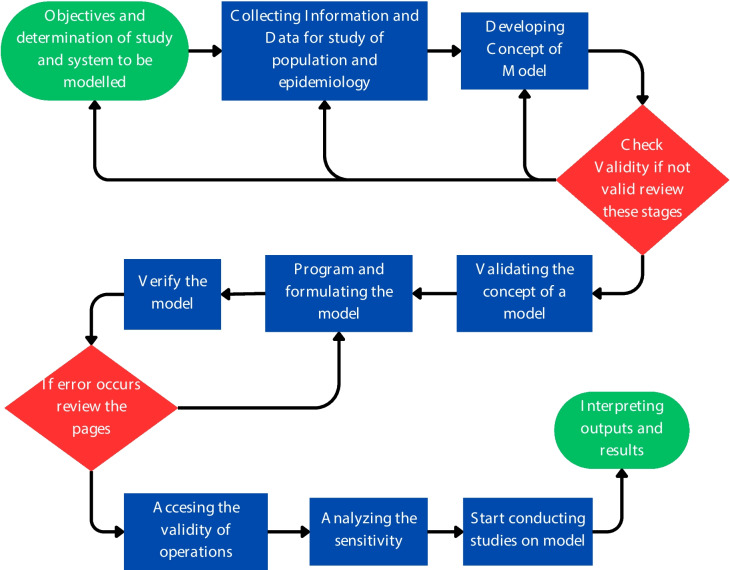
Schematic representation of the process of epidemiological model building which includes determining the study goal, data collection process, model development, model evaluation & validation till the results and output interpretation

In contribution to epidemiology adapted from the work of Taylor [77], Law [78] and Sargent [79] a ten-stage process for developing valid epidemiological disease models was proposed, as depicted in Fig. 2. The first stage involves selecting the specific disease or health issue to be studied and clearly defining the research objectives. Later, Comprehensive data collection is essential, including information on the population under study, the epidemiology of infections, and relevant diseases. This stage ensures that the model is built on accurate and relevant information. Developing a conceptual framework involves outlining the key components and relationships within the epidemiological system being studied. This step lays the foundation for the subsequent modelling process.

Ensuring the validity of the conceptual model involves assessing its logical coherence and alignment with existing scientific knowledge and theories. This stage consists of translating the conceptual framework into a computational model or script that can simulate the dynamics of the epidemiological system. Model confirmation verifies that the implemented model accurately represents the conceptual framework and behaves as expected [80, 81]. Operational validity involves assessing the model's ability to reproduce observed phenomena and produce meaningful outputs under different conditions. Sensitivity testing evaluates the sensitivity of the model to changes in input parameters or assumptions, helping identify potential sources of uncertainty or bias [82, 83]. This stage involves using the validated model to research, such as exploring different scenarios or interventions and analyzing their potential impacts on disease dynamics. Finally, researchers interpret the outputs generated by the model and use them to conclude the epidemiological system under study. Clear communication of findings is essential for informing public health policy and practice [84, 85].

This systematic approach ensures that epidemiological disease models are rigorously developed, validated, and utilized to enhance our understanding of infectious diseases and inform evidence-based interventions. AI has had a significant role in epidemiological modelling throughout the pandemic. Furthermore, the basic epidemiology and model-building process is revised, and the actual implementation of AI and ML-enabled models for COVID-19 are mentioned in further review.

## AI-enabled dynamic models for pandemic

**Fig. 3:**
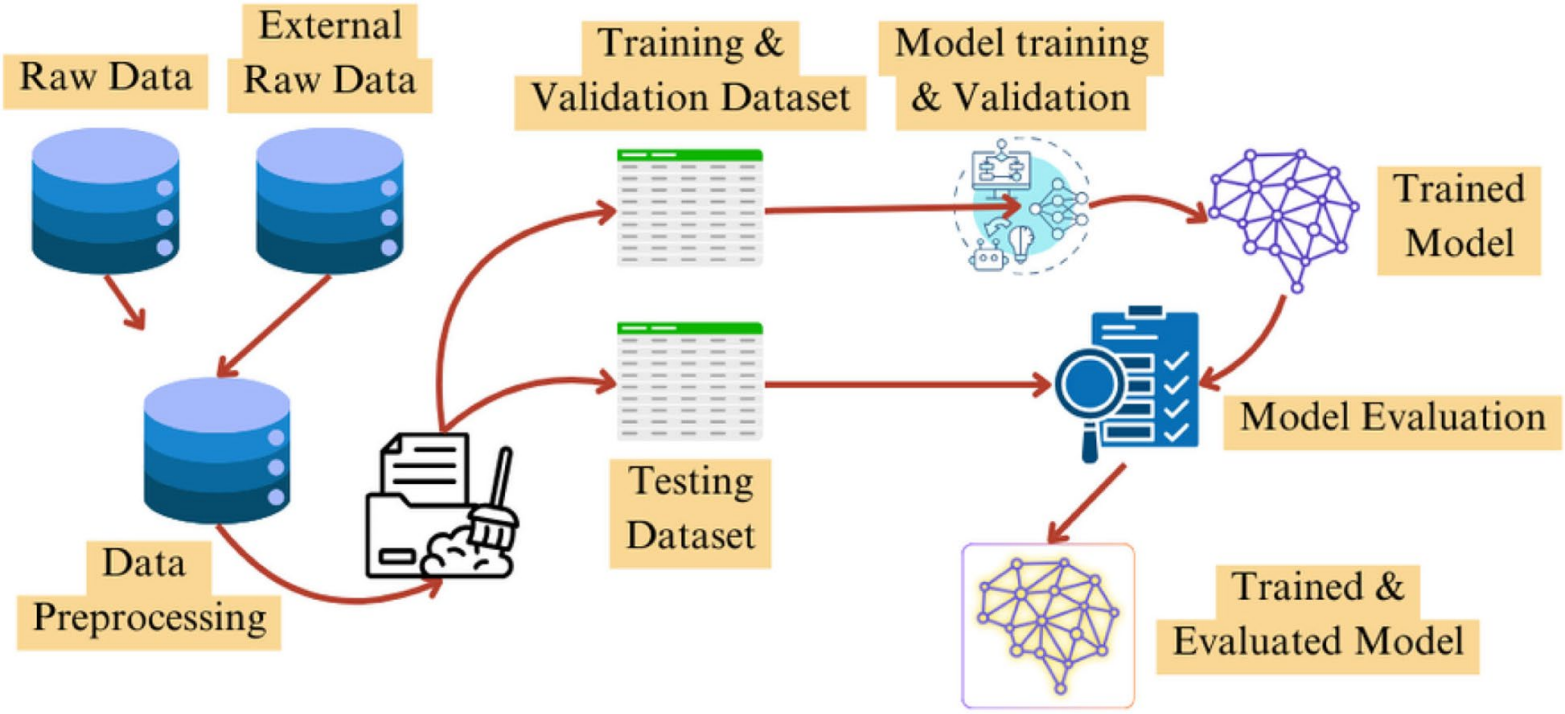
Schematic representation of the process of model creation with AI algorithms which depicts the data collection & transformation, model building, training & testing of model along with evaluation and deployment

The COVID-19 pandemic has sparked an unprecedented utilization of artificial intelligence (AI) and machine learning (ML) algorithms to combat the virus’s spread, comprehend its complexities, and forecast its future trajectory. Dynamic models powered by these algorithms are emerging as potent tools for navigating the ever-changing landscape of the pandemic [86–90]. As machine learning and deep learning algorithms advance, their popularity grows, with anticipated significant positive impacts on the healthcare system. Given the strain on healthcare infrastructures globally due to the pandemic, there’s an opportunity to demonstrate the benefits of integrating AI into healthcare infrastructure. These technologies' adaptability and flexibility allow for effective deployment in combating COVID-19. Using AI and ML technologies aims to improve the accuracy of predictions related to infectious and non-infectious diseases, including COVID-19 [91].The basic process of model building using AI algorithms is illustrated in Fig. 3.

One of the pioneering expert systems in healthcare assistance was the MYCIN system, developed in 1976. This system utilized 450 rules based on medical expert criteria to guide antibiotic selection for bacterial infections, facilitating clinical decision-making and supporting medical staff. The application of AI and ML technologies in data management and decision-making related to contagious disease spread enables the development of rapid diagnostic and screening processes. The World Health Organization (WHO) emphasizes four fundamental aspects crucial for managing health crises like the COVID-19 pandemic: identifying the disease’s cause, understanding transmission modes and vulnerable populations, providing evidence of the infection’s behaviour, and organizing epidemiological models to guide infection prevention, control, diagnosis, and treatment strategies [92–94].

Scientific literature provides insights into various methodologies for developing predictions and processing data in clinical settings. Integrating machine learning (ML) and artificial intelligence (AI) methods and resources enhances the effectiveness of COVID-19 patient screening and diagnostic development. This progress contributes to improving healthcare expert systems, aiding in organizing and planning screening for SARS-CoV-2 carriers. Faster and more precise methods aim to strengthen conventional screening methodologies, reinforcing the overall screening process [95–97]. Image-based AI models for diagnosis of COVID-19 are discussed in Table 1.

**Table 1:** Image-based AI models for early diagnosis of COVID-19 with accuracy and key findings

Title	Modality	ML/AI Technique	Dataset Size	Accuracy	Key Findings	Limitations
Artificial Intelligence Approaches on X-ray-oriented Images Process for Early Detection of COVID-19 [98]	Chest X-ray	ResNet18	4,212 images	100%	AI can effectively identify COVID-19 cases from chest X-rays	• Exclusion of Non-English Studies • Performance Variability • Dataset generalization issues • High computational costs of advanced models
Deep learning for chest radiograph interpretation in COVID-19: A retrospective validation study [99]	Chest X-ray	DenseNet-121	1,121 images	93.4%	Deep learning can achieve high accuracy for COVID-19 detection with moderate-sized datasets	• Generalisability is limited by single institution test sets • RT-PCR used despite 71% sensitivity • No classification is severity or clinical characteristics is included
A Deep Learning Model for Early Detection of COVID-19 Pneumonia from Chest CT Scans [99]	CT scan	VGG19	1,039 images	94.1%	Deep learning can effectively detect COVID-19 pneumonia from CT scans with good accuracy	• Split testing data from the same source was utilised for validation • A reduction in accuracy when using published CT images for external evaluation • The dataset utilised to train AI models is small
COVID-Net: A Tailored Deep Convolutional Neural Network Design for Detection of COVID-19 Cases from Chest X-rays [100]	Chest X-ray	COVID-Net	14,588 images	92.2%	COVID-Net achieves high accuracy and generalizability across diverse datasets	• Validated models using training and validation sources • External CT images from studies that have been published reduced accuracy • Deep learning models are trained with a small amount of data
External Validation of Machine Learning Models for COVID-19 Detection Using Chest Radiographs [101]	Chest X-ray	CNN	37,727 images	84.3%	External validation shows moderate performance of ML models, highlighting the need for further improvement	• Small sample size of 171 participants • No information provided on feature comparison • Models less accurate than RT-PCR t
A deep learning based approach for automatic detection of COVID-19 cases using chest X-ray images [102]	Chest X-ray	CNN, XGBoost	1,121 images	96.6%	The comparison shows XGBoost model achieves higher accuracy than CNNs for COVID-19 detection	• Smaller datasets might limit model generalizability • Variations in image quality and labelling can affect performance • XGBoost models can be computationally expensive
Deep learning for early detection of COVID-19 pneumonia using chest X-rays: A large-scale, multicenter study [103]	Chest X-ray	EfficientNet-B0	14,350 images	93.4%	The deep learning model shows high accuracy for COVID-19 detection in diverse populations	• Not enough samples to train a robust model • Model generalizability is impacted by diverse patient data • There aren’t enough thorough measurements for quantifying diseases

Numerous studies have explored using artificial intelligence (AI) and machine learning (ML) to detect COVID-19 through the analysis of chest X-rays and CT scans. These investigations have demonstrated promising accuracy levels, ranging from 84.3% to 100%, suggesting the potential utility of AI in clinical settings. Deep learning algorithms such as ResNet18, DenseNet-121, and VGG19 consistently exhibited strong performance, although XGBoost also emerged as a viable alternative in one study. While both chest X-rays and CT scans were effective, CT scans generally demonstrated higher sensitivity, indicating the possibility of earlier detection [104, 105]. Deep neural networks have significantly contributed to the detection and treatment of COVID-19, with several notable models such as the Multi-Task Deep Model, CogMol (Controlled Generation of Molecules), COVID-Net, Random Forest Model(RF), Time-Dependent Susceptible-Infected-Recovered(SIR), DarkCOVIDNet, Support Vector Regression(SVR), and Stacking-Ensemble Models [106, 107]. These models have been trained on datasets of 4,895 commercially accessible drugs to identify potential COVID-19 inhibitors, discovered five promising medications, and developed a deep generative model targeting three critical coronavirus protein domains. The Random Forest Model was used to analyze 63 quantitative COVID-19 characteristics from chest CT images, achieving an AUC score of 91% and an accuracy of 87.5%. The Time-Dependent SIR Model was developed to dynamically adjust control strategies by epidemic protocols, while DarkCOVIDNet proposed a method for automatic identification of COVID-19 using raw chest X-ray images, achieving high diagnostic accuracy [100, 108–110].

Support Vector Regression (SVR) and Stacking-Ensemble Models were used for COVID-19 forecasting, with SVR performing the best in terms of forecasting accuracy. Coronet, pre-trained on the ImageNet dataset, achieved an overall accuracy of 89.6%, 93% accuracy and 98.2% recall for COVID-19 patients in a 4-class classification scenario. InstaCovNet-19 employed chest X-ray images to detect COVID-19 and pneumonia, achieving a classification accuracy of 99.08%, with an impressive 99.53% accuracy in differentiating COVID-19 and non-COVID-19 cases [111–113]. These models demonstrate the diverse and powerful ways deep learning has been applied to COVID-19 detection, diagnosis, and treatment discovery, each offering unique contributions to improving the speed and accuracy of identifying and combating the virus.

However, external validation studies emphasize the need for further refinement of ML models to ensure optimal generalizability and performance in real-world scenarios. Additionally, acquiring larger and more diverse datasets is essential to confirm the effectiveness and applicability of these AI-based approaches. Overall, these investigations underscore the promise of AI and ML in facilitating the detection of COVID-19 through radiographic imaging. Nevertheless, ongoing development and validation efforts are crucial before considering widespread clinical implementation. The contribution of ML Models in COVID-19 diagnosis is mentioned in Table 2.

**Table 2:** Machine learning models and their contributions to the pandemic

Name	Contribution
Decompose, Transfer and Compose (DeTraC) [114]	• Framework: CNN-based DeTraC • Pre-trained model: ResNet18 • Performance Metrics: ◦ Accuracy: 95.12% ◦ Sensitivity:97.91% ◦ Specificity: 91.87%
Data-Driven Drug Repositioning Framework [115]	• Method: Utilizing data to investigate medication repurposing • Suggested Drugs Evaluated: More than 6000 • Critical Finding: Inhibitor CVL218 • Emerging Features: Excellent potential for medication repurposing • An advantageous safety profile in rats and monkeys
AI4COVID-19 [116]	• System: AICOVID19 • Input data: Cough or sound signals recorded with cellphones • Performance: ◦ Cough Detection Accuracy: 97.91% • Integration: Domain knowledge from medical experts – 93.56% is the accuracy of COVID-19 detection
Modified Auto- Encoder (MAE) [106]	• Context: Adapted autoencoder • Goal: Emulate COVID-19 transmission dynamics • Data Source: Empirical data from the WHO • Performance: Average error rate of less than 2.5% • Key Observation: Prompt intervention measures significantly reduce infection cases and fatalities

Table 3 discusses various AI frameworks and their contributions to addressing the COVID-19 pandemic. It highlights using machine learning algorithms like decision trees, random forests, XGBoost, and gradient boosting machines to predict COVID-19 diagnoses with over 85% accuracy across all age groups. Additionally, high-performance machine learning models were used to identify crucial biomarkers such as lymphocytes, hs-CRP, and LDH, which were critical in predicting patient mortality with over 90% accuracy more than 10 days in advance. Other models, like Long Short-Term Memory (LSTM) networks, were recommended for forecasting future COVID-19 cases, with some models predicting the pandemic’s end as early as June 2020​.

**Table 3:** AI frameworks and their contributions to the research survey

Names	Contribution
The machine learning algorithms employed in the study encompassed decision trees, random forests, XGBoost, gradient boosting machines (GBM), and support vector machines (SVM) [117]	• Method: Supervised Machine Learning • Goal: Forecast COVID-19 diagnosis • Model: XGBoost algorithm • Result: Prediction accuracy of more than 85%, accurately predicted and detected COVID-19-related features for all age groups
High-performance machine learning Algorithm [118]	• Purpose of Study: Prognostic factors for disease mortality • Source of Data: Blood samples from people in Wuhan, China • Use: Helpful for healthcare organisations in proactive planning and decision-making • Techniques of Machine Learning: Used to find important biomarkers • Key Biomarkers Identified: ◦ Lymphocytes ◦ High sensitivity reactive protein (hs-CRP) ◦ Lactic dehydrogenase (LDH) • Performance: With an accuracy rate above 90%, combined biomarkers predict patient mortality more than 10 days in advance
Deep Learning using LSTM network [119]	• Recommendation: Utilize LSTM networks for deep learning • Goal: Predict future COVID-19 instances • Reference: Long-term LSTM network • Estimation: The epidemic could stop in June 2020
Deep learning: deep neural network (DNN) on the fractal feature of images and convolutional neural network (CNN) methods with the direct use of lung images [120]	• CNN and DNN are compared • The performance metrics are as follows: ◦ CNN: Accuracy:93.2%; Sensitivity:96.1% ◦ DNN: Accuracy:83.4%; Sensitivity:86% • Segmentation Stage: Purpose: Use CNN to identify contaminated tissue in lung images • Results: 83.84% accuracy in identifying the impacted regions • Implication: Committing to track and manage the spread of the disease in impacted areas
Deep neural network (DNN)–based diagnosis solutions, mainly based on convolutional neural networks (CNNs) [121]	• Model: COVID-CAPS • Parameters: Many times, less trainable than in other models • Performance metrics: ◦ Accuracy: 95.7% ◦ Sensitivity: 90% ◦ Reliability: 95.8% ◦ AUC: 0.97 • Progress Methods: Pre-training and transfer learning • Source of Data for Improvement: New dataset created from an outside X-ray image dataset

Table 4 discusses various AI-powered models and their dynamic approaches to resolving challenges posed by COVID-19. It highlights different AI techniques like the Assistant Discrimination Tool, pre-trained network architectures like ResNet-101, and the hierarchical α-Satellite System. These models contributed to COVID-19 management through precise detection and risk evaluation. For example, ResNet-101 achieved a 99.51% accuracy in distinguishing COVID-19 from other conditions, while the α-Satellite system utilized diverse data sources, including social media and demographic statistics, for risk assessments.

**Table 4:** AI and its dynamic model’s algorithms approach in resolvent of COVID-19

Model	Contribution
Method: Assistant Discrimination Tool [122]	• The test set and cross-validation accuracy scores of the technique were 0.9697 and 0.9795, respectively, indicating good accuracy • It proved to have an overall accuracy of 0.9595 and a sensitivity of 0.9512 on an external validation set • These findings demonstrate the tool’s strong ability to discriminate COVID-19 from comparable circumstances in practical settings
Pre-trained networks architecture [123]	• ResNet-101 and Xception outperformed the other nine CNNs in the test when it came to separating COVID-19 from non-COVID-19 situations • With 100% sensitivity, 99.02% specificity, and 99.51% accuracy, ResNet-101 obtained an AUC of 0.994 • Xception likewise attained a 99.02% accuracy, 100% specificity, and 98.04% sensitivity, with AUC of 0.994
α Satellite System [124]	• To assess COVID-19 risk, the α-satellite employs a hierarchical, AI-driven algorithm • Social media, the WHO, and statistics on migration and demographics are just a few of the places it gathers information • An exhaustive risk evaluation is made possible by this varied data integration

The research conducted in this publication involved categorizing works based on models, frameworks, algorithms, and other labels such as architecture, systems, or methodologies. These works aimed to address the challenges posed by the COVID-19 pandemic through the design and implementation of automated models for data analysis using innovative resources and technologies. Specifically, the literature review focused on the COVID-19 Epidempredict initiative, which is a research and development project utilizing various technological resources and tools to facilitate the design and implementation of creative technological solutions. The primary objective of this project is to achieve optimal management of diverse data inputs related to the evolution of COVID-19 in Panama [125–127].

**Fig. 4:**
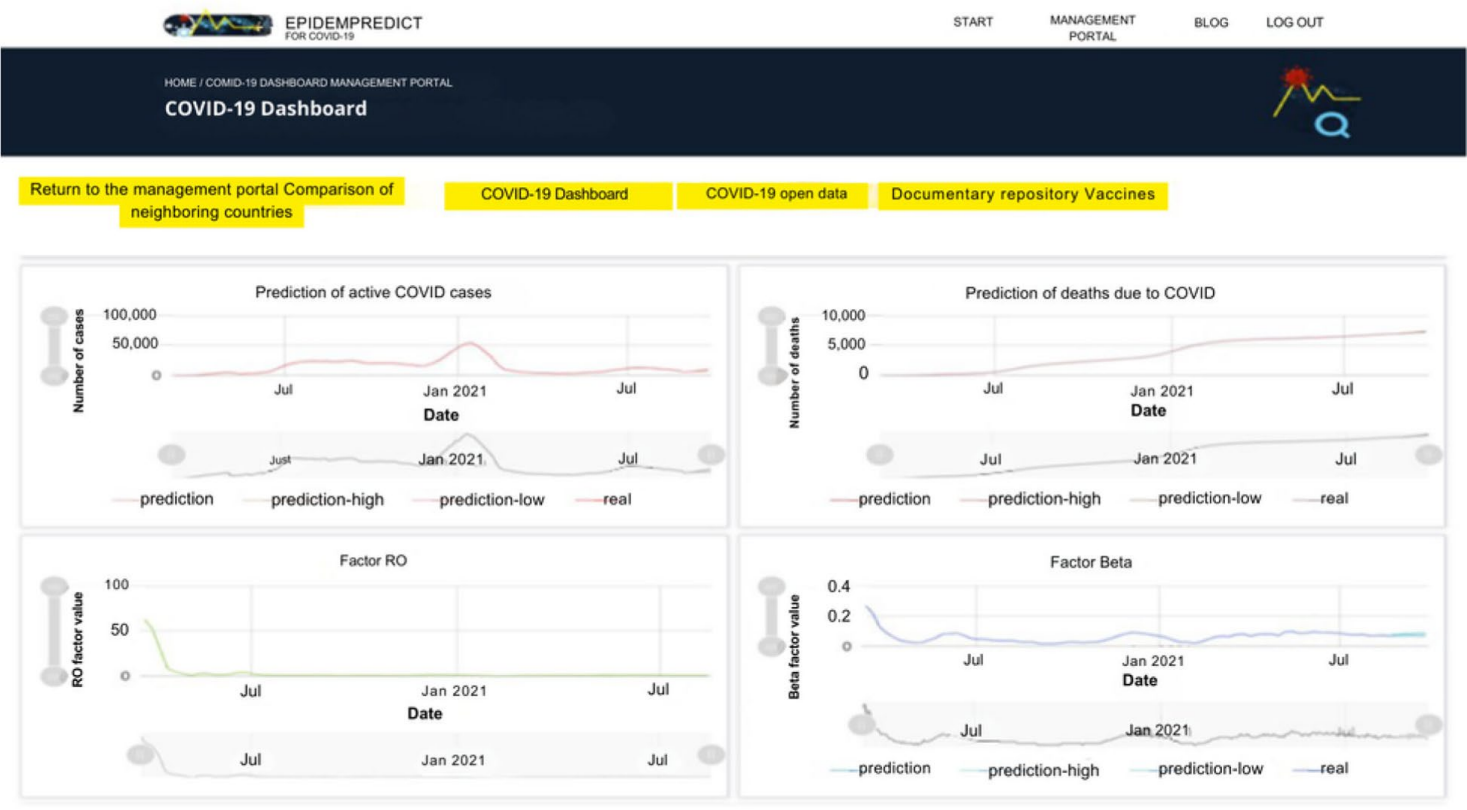
Dashboard designed to make all the project information visible. With permission, this image has been reproduced [128]. Copyright 2021, MDPI

Throughout the development of this project, several sophisticated models were created to enable prediction and optimization tailored to the current demands in terms of data and knowledge regarding the progression of the pandemic in Panama. Additionally, the development of models and algorithms aimed to promote the utilization of simpler, more flexible decision-making procedures. In this scenario, the approach considered the utilization of models that operate on data collected from various sources, including real-time databases such as relational and non-relational ones. To ensure accessibility of all project information, a dedicated website was created with a dashboard as shown in Fig. 4. This dashboard includes a login feature for approved users to protect private health information. Once logged in, users can access various sections, including a comparison of adjacent nations, a COVID-19 dashboard, COVID-19 open data, a document repository, and vaccination information, as well as a breakdown view of the dashboard.

The developed solution incorporates a hybrid model that combines the population dynamics of the SIR model with LSTM recurrent neural networks, employing machine learning algorithms to predict the transmission rate of the virus in Panama. This model utilizes feedback paths between neurons, enabling each neuron to be connected to subsequent ones, past ones, and itself. The EpidemPredict for COVID-19 model adjusts predictions by identifying and detecting changes in the data structure, allowing for predictions that can accommodate fluctuations in key attributes and new data characteristics. The coefficients and results of the model can be adjusted, and data can be consumed and explored through dashboards designed for non-specialized users. Overall, the solution provides direct support for decision-making systems used by health authorities, offering a more accurate and efficient approach to COVID-19 prediction [129].

## AI-powered vaccine development process

Artificial intelligence (AI) has increasingly driven scientific and technological advancements over recent decades. It encompasses a range of tools and methods, including deep learning, machine learning, and neural networks, which serve as a toolbox for addressing complex challenges and driving innovation across various industries. In the field of medicine, AI is utilized in both virtual and physical capacities. The virtual aspect leverages machine learning, particularly deep learning algorithms, and mathematical models, to analyze data, identify patterns, and enhance learning processes [130–132].

**Fig. 5:**
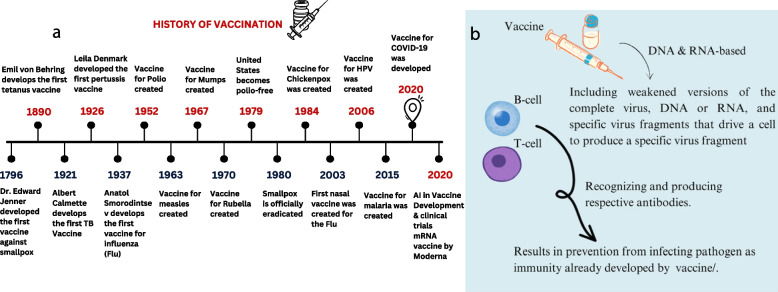
**a** History of vaccinations from the development of first vaccine for Smallpox till the development of vaccine for COVID-19, **b **Vaccination’s ability to stop the spread of viruses imprinting how vaccine response to the host

In healthcare, AI-powered applications have demonstrated significant potential in various domains, including disease diagnosis, treatment optimization, and medical imaging analysis. By harnessing the capabilities of AI, healthcare professionals can gain valuable insights, streamline processes, and improve patient outcomes. Overall, AI is a powerful toolset for tackling complex problems across diverse fields, offering the potential to revolutionize industries and drive progress in numerous areas of human endeavour. The process of the history of the Development of vaccinations is shown in Fig. 5a. Right now, according to this schematic synopsis, we are residing in the present period of vaccine development, which is more advantageous and effective than any other historical era. The two primary areas of Artificial Intelligence in medicine are virtual and physical. Machine Learning and Deep Learning play a significant role in the virtual realm of vaccine development. There are three divisions based on ML algorithms, which include—1] Unsupervised ML, 2] Supervised ML, and 3] Reinforcement ML. Thanks to machine learning and knowledge management algorithms, artificial intelligence has already increased and continues to increase the influence of its techniques in genetics and molecular medicine discoveries. Unsupervised protein–protein interaction algorithms can result in remedial target identification [133]. Modern clustering techniques and adaptive evolutionary algorithms are combined, and the new approach is called “Evolutionary Enhanced Markov Clustering”. Moreover, approaches for developing vaccines are being modified to meet specific nations’ health and economic requirements. This trend directly affects the number and kind of clinical trials carried out and the products under development. The virus’s ability to stop the virus's spread is shown in Fig. 5b.

Vaccines are biological materials that introduce an organism to an infectious agent, which allows the body to build its defence to fend off future infections like COVID-19. This way, preventive measures against contagious diseases and their contraction from another individual are derived. Corebevax, Covaxin, Covishield, Johnson & Johnson, Moderna, Novavax, Sputnik Light, Sputnik V, and Zydus Cadila are approved vaccines and are available to fight against COVID-19. Computational models-based design for therapeutics and vaccines encourages rapid discovery with less cost and effort in a short period [134–137]. Castillo-Hair framed an ML-based next-generation mRNA therapeutics design. This study reviewed Optimus 5-Prime, the convolutional neural network model deployed to train the experimental data. It was shown that in general, the 5’UTRs have similar effects when combined with various protein-coding sequences [138]. One of the prominent ways of Drug Discovery by analyzing the pattern of expressed genes is reverse vaccinology. The method surveys episodes and antigens to find the possible targets without doing “wet lab” experiments. Edison designed a coronavirus vaccine using the reverse vaccinology technique integrated with Machine Learning. The following experimental work is done using Vaxign an in-house vaccine design tool for the prediction of vaccine candidates. This study predicted six proteins from the proteome, which acted as adhesins and ensured viral adhesion and host invasion. However, nsp3 was more conserved among the SARS family than 15 other variants of CoronaVirus [139, 140].

## Implementation of COVID-19 vaccination using AI

A vaccine typically contains several components such as an agent resembling a disease-causing microorganism, Surface proteins of the microorganism, and Toxins produced by the microorganism which are discussed as follows. An agent resembling a disease-causing microorganism: This agent is often manufactured from weakened or inactivated microbes, which stimulate the body’s immune system to recognize and mount a defence against the microorganism. Surface proteins of the microorganism: These proteins play a key role in the interaction between the microorganism and the host’s cells. By including surface proteins in the vaccine, the immune system can generate antibodies specific to these proteins, protecting against infection [141–143].

Toxins produced by the microorganism: Some vaccines may also contain inactivated toxins produced by the microorganism. These toxins can trigger an immune response and help the body recognize and defend against the harmful effects of the microorganism. Vaccines can be administered as preventive measures to protect individuals from infection or as therapeutic measures to treat existing infections. They represent one of the most significant public health interventions globally, particularly in the context of the COVID-19 pandemic caused by the highly contagious SARS-CoV-2 virus [144–146].

Developing vaccines and antiviral treatments against SARS-CoV-2 is crucial due to the virus’s rapid spread and relatively high fatality rate compared to other viral infections. However, vaccine development typically requires a significant amount of time, often ranging from several years to decades. The vaccine trial process involves various steps that must be followed systematically to ensure safety and efficacy. In recent years, the pharmaceutical industry has increasingly utilized artificial intelligence (AI) to streamline vaccine development processes. AI algorithms can analyze vast amounts of data, identify patterns, and expedite decision-making. For example, Pfizer employed AI during vaccine trials to analyze data efficiently and ensure the vaccine met regulatory standards. Additionally, AI tools such as Smart Data Query (SDQ) have helped analyze data rapidly, accelerating the vaccine development process. Table 5 shows vaccines developed by various manufacturers with the use of AI. Overall, AI plays a crucial role in modern vaccine development, helping researchers expedite the discovery and production of vaccines to combat infectious diseases like COVID-19 [76, 147, 148].

**Table 5:** Vaccines developed by various manufacturers with the use of AI

Sr. No	Vaccines with manufacturer	Use of AI/ML
1	BNT162b2/Comirnaty BioNTech/Pfizer/Fosum Pharma Li, [149]	• Release of data 22 h after it became effective • SDQ technology improved vaccination yield • The predicted freezer temperatures from ML/AI • Lipid nanoparticles optimised using supercomputing
2	BBV152/Covaxin Bharat Biotech Kumar and Veer, [150] Malhotra, [151]	• Necessitates storage at 2–8 °C • IoT technology makes real-time monitoring possible • Temperature fluctuations are detected using sensors • Notifies when the next batch is prepared
3	AZD1222/Covishield AstraZeneca/Oxford University Sharma, [152], Kumar and Veer, [150]	• The AI discovered biomarkers 30 times faster • ML expedites the evaluation of tissue samples • Real-time data monitoring is made possible via IoT • Sensors improve patient enrolment and identification
4	mRNA-1273 Moderna Asada, [153]	• AI facilitates the synthesis of mRNA sequences; mRNA output rose from 30 to 1,000 per month • Made possible by AI and robotic automation

Cohen notes in this assessment that the pharmaceutical sector has incorporated AI into the process of developing vaccines, therefore cutting down on the time required to produce a vaccine prototype that can be tested on humans. AI and other bioinformatics technologies have been used by American startup Moderna to examine and fix millions of data points for inaccuracies. Moderna was among the first to provide effective COVID-19 immunizations because of this technology, which has considerably decreased the time spent on data sets [154]. Additionally, Moderna, a leader in COVID-19 immunization, is using AI to hasten the creation of mRNA sequences. Chief Data Officer and AI Officer Dave Johnson describes how Moderna was able to generate more than 1,000 mRNAs every month with robotic automation and AI algorithms. As evidence of the quick progress in AI-driven vaccine research, AstraZeneca, a major partner in the Covishield vaccine, also employs AI in medication discovery and development. Leading healthcare provider AstraZeneca is known for incorporating artificial intelligence into its drug-making process to expedite, lower costs, and increase safety. The company’s Covishield, created by Oxford-AstraZeneca, detects biomarkers 30% quicker than human pathologists by utilizing knowledge graphs and image analysis. The COVID-19 vaccine Covaxin from Bharat Biotech needs to be stored at 2–8 °C in a thermoregulated environment. A dependable storage system is ensured using IoT-based sensor technology, which uses sensors to detect temperature changes and notify the next shipment of vaccinations [155].

**Fig. 6:**
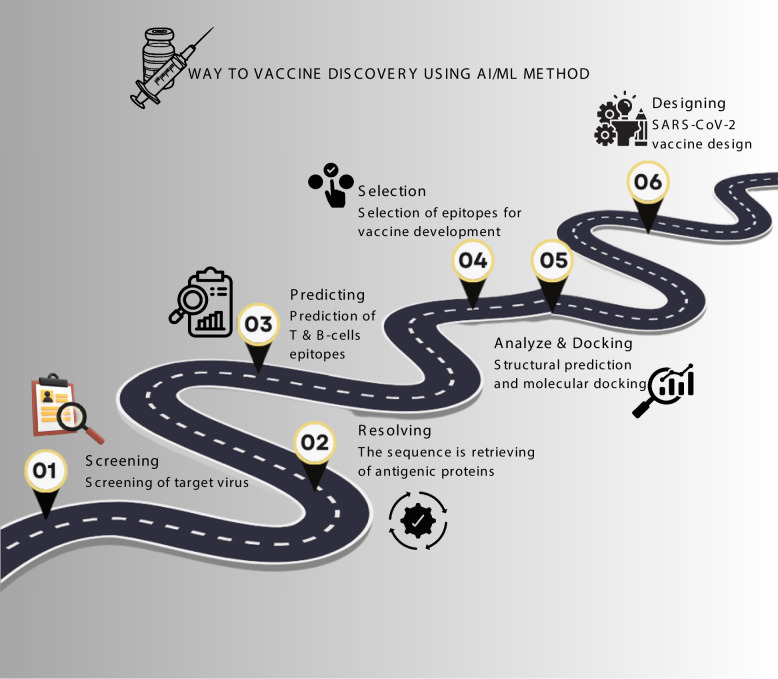
Schematic representation of the roadmap for vaccine discovery by AI and ML method, how AI/ML optimize the process of screening, resolving, predicting analyze & docking, selecting and finally the designing of vaccine

In Fig. 6, many possible vaccine candidates are screened, and the most promising ones are then chosen for further processing. The most promising candidates are chosen for additional research after being evaluated to see how effectively they might function as a vaccine. Lastly, the chosen candidates undergo testing on both people and animals to ensure their efficacy and safety. Traditional methods of vaccine discovery are time-consuming and costly, as AI and ML can efficiently screen large molecules for effective vaccines, saving valuable time and money. AI and ML are being utilized to analyze promising candidates in vaccine candidates, enabling researchers to concentrate on the most promising ones. Through their assistance in the initial development of novel vaccine candidates, AI and ML are addressing the difficult issue of creating effective vaccinations [90].

### ML and AI technology in SARS-CoV-2 screening and treatment

The Coronavirus illness is one of the diseases that AI and ML are being used to screen for and identify. These technologies are being utilized to improve the screening and diagnostic process, including clinical blood sample data, X-rays, and Computed Tomography (CT). These conventional techniques have, however, performed only mediocrely during the epidemic. Research has indicated that radiologists can use Deep Convolutional Networks (Resnet-101) as an adjuvant tool to help with this. The study demonstrated that the convolutional neural network (Resnet-101) produced amazing performance, attaining 86.27% accuracy and 83.33% specificity, on 1020 CT scans of 108 Covid-19 infected patients and viral pneumonia of 86 patients using an expert system utilizing AI and ML. It is anticipated that this novel method of diagnosis and screening will transform healthcare and prevent deaths during the pandemic [95, 96].

Developing a new automatic COVID-19 detection model based on deep learning algorithms represents a significant advancement in medical diagnostics. This model utilizes raw chest X-ray images from a dataset comprising infected patients, as well as cases of no-findings and pneumonia. The remarkable performance accuracy of the model, with a binary class accuracy of 98.08% and a multi-class accuracy of 87.02%, underscores its potential utility in clinical settings. In addition to leveraging deep learning for image analysis, researchers have also identified four essential medical features GHS, CD3 percentage, total protein, and patient age—to enhance diagnostic accuracy. These features are used with a Support Vector Machine (SVM) as the primary classification model. The robustness of this approach is demonstrated by high AUROC values of 0.9996 and 0.9757 in training and testing datasets, respectively, particularly in predicting patients in critical or severe conditions. How AI and ML technologies collaborate in SARS-COV2 screening is depicted in Table 6.

**Table 6:** ML and AI technology in SARS-COV-2 screening

Reference	Types of data	AI Technique	Validation method	Sample size	Accuracy
Ardakani et. al. [123]	Clinical, Mammographic	Deep Convolutional Neural Network ResNet-101	Holdout	86 CT scans of patients with viral and atypical pneumonia, 1020 CT scans of 108 individuals with laboratory-confirmed Covid-19	Accuracy: 99.51% Specificity: 99.02%
Ozturk, T. et.al. [156]	Clinical, Mammographic	Convolutional Neural Network DarkCovidNet Architecture	Cross-validation	127 X-ray pictures showing 500 no-findings, 500 pneumonia cases, and 43 female and 82 male positive cases	Accuracy: 98.08% on Binary classes Accuracy: 87.02% on Multi-classes
Sun, L et. al. [156]	Clinical, laboratory features, Demographics	Support Vector Machine	Holdout	PCR kit-positive 336 individuals, 26 critically ill or severe cases, 310 non-critical cases, and one additional linked illness29 diabetes, 17 coronary diseases, 79 hypertension, 7 tuberculosis history, and 17	Accuracy: 77.5% Specificity: 78.4%
Wu, J. et. al. [122]	Clinical, Demographic s	Random forest Algorithm	Cross-validation	253 samples total, gathered from various sources, were taken from 169 individuals who were suspected of having Covid-19	Accuracy: 95.95% Specificity: 96.95%

The application of an expert system in healthcare, such as the developed model, holds promise for improving diagnostic accuracy and reducing disease spread, particularly in rapidly evolving situations like the COVID-19 pandemic. However, there remains a need for further research to refine and validate these models for real-world deployment [157]. One area of improvement suggested by the study is the development of hybrid classification methods that integrate clinical, mammographic, and demographic data. Such hybrid approaches may offer enhanced diagnostic capabilities and facilitate broader adoption of AI-driven diagnostic tools in clinical practice.

### ML and AI technology in SARS-Cov-2 contact tracing

Contact tracing plays a crucial role in preventing the transmission of COVID-19 by identifying and treating individuals who have recently been in contact with infected patients. Digital contact tracing methods, leveraging technologies such as Bluetooth, GPS, and social graph analysis, have been implemented in many affected countries to track and trace potential contacts more efficiently. These digital applications collect personal information from users, which is then analyzed using machine learning and artificial intelligence techniques to identify and notify individuals who may be at risk based on their recent contact history. Digital contact tracing apps offer several advantages over traditional methods, including real-time operation and faster response times, which can help contain outbreaks more effectively and reduce the spread of the virus. However, there are also concerns regarding privacy, data security, and compliance with regulations. Some countries have passed emergency laws or violated privacy laws to implement contact tracing programs, raising questions about transparency, data control, and the protection of individuals’ rights [158, 159].

To improve the efficiency of contact tracing, some researchers propose using graph theory to analyze data on infectious disease outbreaks, such as animal disease shipments between farms, to predict the potential spread of infections. However, it is essential to address privacy concerns and ensure that individuals’ data is protected and used responsibly. Overall, while digital contact tracing can be a valuable tool in controlling the spread of COVID-19, it is essential to strike a balance between public health measures and individual privacy rights. Transparency, data security, and adherence to privacy regulations are crucial for building public trust and ensuring the effectiveness of contact-tracing efforts [160].

### AI technology in SARS-CoV-2 prediction and forecasting

The mentioned studies highlight the utilization of various machine learning and artificial intelligence techniques to forecast and predict the spread of COVID-19 in different regions, including Brazil, Canada, India, and globally. These models employ innovative approaches such as stacking ensemble with support vector regression, XGBoost classifier, deep learning algorithms like LSTM networks, and hybrid wavelet autoregressive integrated moving average models. In Brazil, a stacking-ensemble model with support vector regression was developed to forecast COVID-19 cases in ten Brazilian states, enhancing short-term forecasting capabilities. In recent works, a unique model utilizing the XGBoost classifier was proposed, focusing on clinical and mammographic factors, with significant accuracy in predicting and classifying COVID-19 patients based on key features such as high-sensitivity C-reactive protein, lymphocyte, and lactic dehydrogenase (LDH) [111, 161].

Similarly, a deep learning algorithm in Canada was employed to predict the SARS-CoV-2 epidemic, with forecasts indicating an endpoint around June 2020. This model’s applicability was validated using data from Johns Hopkins University, providing valuable insights for policymakers and healthcare experts. Additionally, real-time forecasting models incorporating wavelet-based forecasting and autoregressive integrated moving average-based time series were developed for several countries, including India, the United Kingdom, Canada, South Korea, and France, serving as early warning systems for pandemic outbreaks.

In India, machine learning and artificial intelligence applications have been utilized to predict and forecast COVID-19, demonstrating varying levels of accuracy ranging from 0.87% to 90% across different techniques such as Support Vector Regression, stacking ensemble, XGBoost classifier, LSTM networks, and hybrid wavelet autoregressive integrated moving average models. Table 7 shows the contribution of AI in SARS-CoV-2 prediction & forecasting. Overall, these studies underscore the importance of leveraging advanced computational techniques to address the challenges posed by the COVID-19 pandemic, providing valuable insights for decision-makers and healthcare professionals in managing and mitigating the spread of the virus.

**Table 7:** AI technology in SARS-CoV-2 prediction and forecasting

**Tool Name**	**Functionality**	**Impact on Vaccine Development**	**Limitations**	**Integration**	**Regulatory Considerations**	**Scalability & Generalizability**	**Transparency & Explainability**
Oracle Cerner Enviza [162]	Real-time data analysis, streamlined data management	Faster trial completion informed decision-making	Requires robust data infrastructure, potential vendor lock-in	Moderate (integrates with existing clinical research platforms)	Compliance with data privacy and security regulations	Limited to specific clinical research platforms	Moderate
XtalPi’s AI platform [163]	Protein structure analysis, target identification	Accelerated identification of vaccine targets, improved vaccine design	Relies on accurate protein structure data, and may require further validation	Moderate (integrates with existing protein structure databases)	Moderate (requires specific data governance and access protocols)	Limited to protein-based vaccines	Low
IQVIA’s Orchestrated Clinical Trials platform [164, 165]	Remote patient monitoring, improved data collection	Reduced participant burden, efficient data capture, minimized exposure risk	Requires compatible devices and infrastructure, potential privacy concerns	Moderate (integrates with various wearable devices and apps)	Compliance with data privacy and security regulations, ethical considerations for remote monitoring	Applicable to various clinical trials with remote monitoring needs	Moderate
Insilico Medicine’s AI platform [166]	Efficacy prediction, candidate prioritization	Prioritized promising candidates, saved time and resources	Relies on accurate simulations and data, interpretability limitations	Moderate (integrates with existing clinical trial data and simulation platforms)	Moderate (requires adherence to regulatory guidelines for AI-driven predictions)	Potentially applicable to diverse vaccine development pipelines	Low
BenevolentAI’s AI platform [163]	Target and epitope identification, antibody response prediction	Accelerated vaccine development, prioritized candidates with strong immune response	Limited to specific datasets, requires integration with other data sources, limitations in accuracy and generalizability	Moderate (integrates with various biological and clinical data sources)	Moderate (requires adherence to data privacy and security regulations)	Applicable to diverse vaccine development pipelines	Low
Exscientia’s AI platform [167]	Vaccine candidate identification	Identified promising malaria vaccine candidate, currently in trials	The early stage requires further validation and clinical testing	Moderate (integrates with existing drug discovery and development platforms)	Moderate (requires adherence to regulatory guidelines for AI-driven drug discovery)	Potentially applicable to diverse vaccine development pipelines	Low
Freenome’s AI platform [168]	Early complication prediction	Potential for personalized treatment strategies, requires further research and clinical validation	Limited data on long-term impact, potential ethical concerns regarding early intervention	Low (limited integration with existing clinical workflows)	Compliance with data privacy and security regulations, ethical considerations for early intervention	Potentially applicable to diverse disease management	Low
Atomwise’s AI platform [169]	Virtual screening for small molecule drug candidates	Identified potential vaccine adjuvants, accelerated pre-clinical development	Limited to specific molecule types, requires further optimization for vaccine development	Low (limited integration with existing vaccine development platforms)	Compliance with data privacy and security regulations	Limited to small molecule-based vaccines	Low
DeepMind’s AlphaFold [170]	Protein structure prediction	Improved understanding of viral proteins, aided in vaccine design and development	Limited to specific protein types, requires further validation and refinement	Moderate (integrates with existing protein structure databases and modeling tools)	Moderate (requires adherence to data privacy and security regulations)	Potentially applicable to diverse protein-related research areas	High

## AI in clinical trials of the vaccine

The development of vaccines is a complex and time-consuming process, with testing being one of the most critical and resource-intensive stages. However, the integration of computational methods and artificial intelligence (AI) strategies has significantly accelerated vaccine development processes. AI facilitates the rapid classification of new viruses by identifying inherent genomic architecture and similarities with known infections. It assists in quickly identifying viable candidates for clinical trials by analyzing digital medical data and screening potential individuals. Some researchers have reported reducing the time between molecules coming to market from 10 years to one year using AI tools like Smart Data Query (SDQ) to evaluate clinical trial data in less than twenty-four hours.

Regulatory agencies worldwide divide the vaccine development process into preclinical and clinical trials, with guidelines provided by organizations such as the European Medicines Agency (EMA), the US Food and Drug Administration (USFDA), and the World Health Organization (WHO). Clinical trials typically progress through four phases: Phase I involves assessing the vaccination schedule, model, and dosage in healthy participants; Phase II evaluates safety and immunogenicity in a small group; Phase III tests safety and effectiveness on a larger population; and Phase IV monitors effectiveness and safety post-licensing [171, 172]. While AI tools cannot replace the rigorous procedures of preclinical and clinical trials, they can assist in patient selection for trials, prediction of therapeutic targets, lead compound prediction, and medication adherence monitoring. For example, AI tools like AICure help monitor medication intake in phase II trials, increasing patient adherence and trial success. Various Real-World applications of AI in response to pandemic are also discussed. AI plays a crucial role in various aspects of vaccine development, including trial planning, recruitment optimization, risk monitoring, toxicity prediction, and drug adherence monitoring, ultimately contributing to the acceleration of vaccine development processes [173, 174].

## Real-world applications of AI in pandemic preparedness

AI has swept away many of the health-related sectors and areas lately, especially during the COVID-19 situation. Among them, early detection systems are a significant aspect. This system uses AI algorithms to scan through sources, such as social media and healthcare records, to discover signs indicating a probable outbreak. For instance, BlueDot used a combination of natural language processing and machine learning to identify early indicators of COVID-19. In prognostic modelling, AI models predict the growth of infectious diseases on the basis of population density and travel patterns. Out of many, Metabiota is an important step in predicting outbreaks and providing a quantified impact assessment. AI has also made it much faster to Explore Therapeutics by analyzing big data for spotting potential treatments [175–179]. For Example, Benevolent identifies drugs for several diseases using AI. AI can be applied in the design of vaccines to analyze genomic data and design customized vaccines. For example, Insilico Medicine applies AI to identify novel targets for novel vaccine development. AI is also applied in monitoring and surveillance to detect and respond to emerging patterns in real time. Health Map aggregates multi-source information in tracking and predicting outbreaks, thus optimizing the ability to monitor the disease. However, the AI also optimizes medical supplies through supply chain optimization- an efficient and timely delivery process. IBM Watson Supply Chain is one notable example which uses AI in logistics management. Telehealth and Chatbots that function with AI ensure critical health information, symptom assessment, and telehealth service provision. For instance, Ada Health uses AI-driven symptom checkers to provide tailored health assessments, making it easier to seek healthcare, especially at a time when such assistance is needed the most. Such applications demonstrated how AI approaches critically in early diagnosis, treatment development, vaccine formulation and operational efficiency [180, 181].

## Challenges and ethical considerations of AI in pandemic response

Robust ethics and risk assessment protocols are necessary to ensure that COVID-19 ethically uses AI. However, implementing these in a crisis is not simple, especially when new technology is being used at a pace and scope never seen before. For example, to successfully lead policy initiatives, forecasting models must be available from the early stages of the spread of sickness and use all available data. The imminence of a catastrophe highlights the shortcomings of the extant, still-developing frameworks for risk assessment and ethics concerning the application of AI. The COVID-19 pandemic exemplifies how rapidly new technologies may need to be implemented to save lives during catastrophes. But this hurry also makes it more likely that risks and moral conundrums will arise and will be harder to resolve. Rather than overlooking ethics, we need to immediately learn how to put ethics into practice. The COVID-19 crisis has prompted politicians, technologists, ethicists, and medical professionals to consider the application of ethics in the use of AI for social good [181, 182]. Ethical guidelines for AI applications in healthcare aim to assist developers, users, and regulators in enhancing and overseeing the development and deployment of these technologies. The basic principles of human dignity and inherent value serve as the foundation for ethical percepts. Governments should outlaw or restrict their usage if AI or other technologies violate human rights, do not follow other rules and laws, or are introduced in inappropriate situations. More data protection legislation and regulatory frameworks are needed to direct the use of AI technology. Respect for human rights is crucial, and machine learning may make protecting and enforcing them easier [183].

The relationship between ethical standards and human rights provides a strong foundation for governments, international organizations, and private actors. Ethics supplement and grow upon human rights accords, balancing competing ideals. Morally acceptable choices must consider all relevant ethical concerns, different viewpoints, and a fair decision-making process. The COVID-19 outbreak has led to control measures like lockdowns and large-scale testing. Regulatory authorities are crucial in defining policies that encourage collaboration among various stakeholders to prevent disease [184, 185].

### Lack of standard datasets

The lack of standard datasets poses a critical challenge in ensuring the trustworthiness of AI and big data platforms and applications in combating the COVID-19 virus, as many proposed algorithms and platforms are not tested using the same dataset. Government, large corporations, and health organizations like WHO and CDC are working together to develop high-quality and large datasets for COVID-19 detection. These entities provide various data sources, such as X-ray and CT scans from hospitals, satellite data, personal information, and self-diagnosis app reports. The CORD-19 dataset, led by Georgetown’s Center for Security and partners like the Allen Institute for AI, Chan Zuckerberg Initiative, Microsoft Research, and National Institutes of Health, exemplifies this collaboration. Alibaba DAMO Academy has collaborated with Chinese hospitals to develop AI systems for COVID-19 detection, with an impressive 96% accuracy within 20 s [186, 187]. Other initiatives include managing public datasets of COVID-19 medical images and a global collection of open-source projects on the virus.

### Privacy and security challenges

The COVID-19 pandemic has highlighted the need for a thorough investigation into the security and privacy of personal data. The Zoom video conferencing app scandal highlights the need for privacy and security measures. Authorities may request personal information like GPS location, CT scans, and daily activities to control the situation and make informed decisions. However, people often resist sharing their personal information, highlighting the trade-off between privacy and performance. Various technologies are available to address these privacy and security issues, and these could serve as research directives for future research [188, 189].

### Model development and validation

Model development and validation represent critical stages in harnessing the power of artificial intelligence (AI) for pandemic response. Amidst the urgency of rapidly evolving public health crises, such as pandemics, the development of AI models faces unique challenges. These challenges stem from the need for models to accurately capture the complex dynamics of infectious diseases while accommodating the variability inherent in real-world data. Developing AI models that provide timely and reliable insights requires a delicate balance between speed and accuracy. Moreover, the generalizability of these models across diverse populations and geographical regions presents a formidable hurdle. Ensuring that AI models trained on data from one region or demographic group can effectively generalize to other contexts is essential for their widespread applicability.

### Data harmonization and integration

Data harmonization and integration are fundamental in leveraging artificial intelligence (AI) for effective pandemic response. These processes involve consolidating and unifying data from disparate sources to create a comprehensive and coherent dataset. However, achieving successful harmonization and integration presents several challenges. One significant challenge is the heterogeneity of data sources. Data collected during a pandemic response may come from various sources, such as healthcare facilities, public health agencies, academic institutions, and community organizations. These sources often use different data formats, structures, and terminology, making it difficult to merge them seamlessly. Standardizing data formats, definitions, and coding systems is essential to overcome this challenge and ensure interoperability between different datasets [110, 156].

## Future prospects

The need to investigate the impact of AI in advertising and reducing pandemics, including new dangers like Dengue, Salmonella, and Monkeypox, is growing as we look beyond the past COVID-19 pandemic. AI can significantly improve our reaction and readiness plans. According to the literature, many AI/ML-based models have proven successful in forecasting pandemic futures, including infection or transmission rates. AI systems could be used for pandemic prevention and response, including early disease detection, predictive modelling, vaccine development, resource allocation, drug discovery, contact tracing and public health communication. The availability of genuine patient data exchange and machine learning approaches help in building models [190–192]. AI is beneficial in predicting virus propagation, relieving pressure on medical professionals and the rush to develop a treatment for COVID-19. Few-shot and transfer learning could be potential gains, aligning with infectious disease management and highlighting AI’s pivot role in global public health. Large Language models like ChatGPT or Google Bard could also be explored for future pandemic outbreaks by disseminating accurate information, answering questions about prevention, symptoms, testing, and vaccination, and providing crisis helplines and mental support [193, 194]. In a pandemic like COVID-19, AI generative models can also help detect and correct misinformation, aid in research and drug discovery, support remote patient monitoring, engage citizens in policy discussions, design awareness campaigns, and enhance healthcare worker training. The SARS-CoV-2 pandemic has accelerated the adoption of AI, ML, and robots for new functions, potentially leading to advanced Pandemic assessment, creating new career openings and essential for the healthcare industry. There is no direct link between AI exposure and employment, but more engagement with AI is associated with faster employment rates in jobs with substantial technology use. Automation in laboratories and supply chains is becoming an overlooked opportunity, and AI advancements can help improve drug development accuracy and reduce time. Additional funding and advancements in AI technology could be crucial in combating future pandemics. As AI is mainly data-driven, ensuring the authenticity and privacy of datasets may be challenging. Despite these challenges, AI remains a cutting-edge tool for battling the COVID-19 epidemic and can potentially change the competent- health care division [195–198].

## Conclusion

This review explores key aspects of epidemiological modelling, such as descriptive, analytic and experimental modelling, that are essential for vaccine development and pandemic responses. The Role of AI in Epidemiology is elaborated and discussed to compensate for the epidemiological methods. This review also examined the various steps in model building with the AI-enabled Dynamic Models. A significant emphasis is placed on how these algorithms contribute to managing pandemic situations, with specific insights into AI/ML models used to address challenges during the COVID-19 pandemic. The use of AI tools during the pandemic highlights the crucial role AI plays in responsibly and efficiently managing health crises. Artificial Intelligence (AI) has revolutionized vaccine development, accelerating the search for life-saving vaccines and simplifying clinical trials. AI/ML methods have been used in various applications, including screening, treatment, contact tracing, prediction, and forecasting. This has led to the development of personalized immunizations and customized vaccines for individual genetic compositions. However, the extensive use of AI in pandemic response raises ethical concerns. Data privacy must be protected, algorithmic biases mitigated, transparency and accountability ensured, and individual autonomy and informed consent maintained. AI has improved early detection and reaction processes, enabling worldwide collaboration via monitoring networks across geopolitical boundaries. Real-world applications like ProMED-Mail and BlueDot demonstrate the real influence of AI in anticipating epidemics and guiding response operations. A cautious approach to AI use, balancing proactive readiness and individual rights, is still required. The continued investigation, ethical use, and integration of AI technologies illuminate the path towards a more resilient global health environment. While AI models may not be entirely efficient, future developments in AI and machine learning will lead to increased efficiency. They may be useful in future health crises like the COVID-19 pandemic.

## Data Availability

Not applicable.
